# Testicular tissue bank: ten years of testicular tissue cryopreservation in Croatia

**DOI:** 10.3325/cmj.2025.66.71

**Published:** 2025-02

**Authors:** Marija Ćapin Vilaj, Monika Trupinić, Ante Jug, Dinko Hauptman, Zoran Zimak, Željko Kaštelan, Davor Ježek

**Affiliations:** 1 Department of Transfusion Medicine and Transplantation Biology, University Hospital Centre Zagreb, Zagreb, Croatia; 2 University of Zagreb, School of Medicine, Zagreb, Croatia; 3 Department of Urology, University Hospital Centre Zagreb, Zagreb, Croatia; 4 Department of Histology and Embryology, University of Zagreb School of Medicine, Zagreb, Croatia; 5 Centre of Excellence for Reproductive and Regenerative Medicine, University of Zagreb, School of Medicine, Zagreb, Croatia

## Abstract

The Testicular Tissue Bank has been operating at the University Hospital Centre (UHC) Zagreb since 2013. It aims to cryopreserve testicular tissue from patients with azoospermia. If spermatozoa are found in the collected tissue, a combined procedure known as testicular sperm extraction (TESE)/intracytoplasmic sperm injection (ICSI) is performed. During the last 10 years, our bank has deposited samples from 443 patients collected either by conventional TESE or microsurgical TESE procedure. Among them, 9% were from oncological patients whose samples were stored to preserve fertility. According to pathohistological analysis, 17% of patients were diagnosed with complete spermatogenesis or hypospermatogenesis, 11% with spermatogenic arrest, 48% with mixed atrophy of seminiferous tubules, and 24% with Sertoli cell-only phenotype or tubular fibrosis. Overall, the presence of testicular spermatozoa was found in 58% of patients, which makes them suitable for the ICSI procedure. In 21 out of 59 patients (36%) who underwent a salvage TESE, the outcome was different than that of the first spermatozoa retrieval attempt. Considering that the male factor is one of the leading causes of infertility, the results of Testicular Tissue Bank activity confirm its important role in improving the demographic picture of Croatia.

## Testicular tissue banking

Tissue banking was established in the early twentieth century to ensure an immediate availability of tissues for clinical needs. Thus, methods for preserving live tissue for extended periods of time for future use were developed (1). Testicular tissue cryopreservation (TTC) is a dependable procedure and frequently the only option for preserving fertility in male patients with azoospermia dealing with cancer, other medical conditions, or social factors. An estimated 15% of all couples attempting to conceive are affected by infertility, and half of infertility issues have a male component (2). The Testicular Tissue Bank at the Zagreb University Hospital Center was established in 2013, when it was also registered in the Assisted Reproduction Registry of the Republic of Croatia.

The preferred method for preserving male fertility is the cryopreservation of mature spermatozoa. Spermatozoa can be obtained immediately after ejaculation or, more invasively, via various microsurgical methods. In azoospermic patients, no spermatozoa are present in the ejaculate, so these cells can be obtained surgically from the epididymis by microepididymal sperm aspiration (MESA) (3,4) or percutaneous sperm aspiration (PESA) (5). Namely, azoospermia can be caused by an abnormality or blockage in the epididymis or vas deferens. This is referred to as obstructive azoospermia (OA). In these cases, although spermatozoa are produced in the testes, due to the obstruction, they are completely absent in the ejaculate (6). Both MESA and PESA are used to retrieve spermatozoa from the epididymis when their transport between the epididymis and urethra is impaired. In the case of MESA, a microsurgical skin incision is made, and a needle is used to aspirate the fluid from the epididymal duct. In PESA, spermatozoa and epididymal duct fluid are aspirated by passing a thin needle through the scrotal skin. Also, OA patients may undergo testicular sperm aspiration (TESA), in which tissue/spermatozoon is aspirated directly from the testicle (7). This method saves a number of healthy spermatozoa, which can be frozen for later procedures. While MESA, PESA or TESA enable the retrieval of any spermatozoa, testicular sperm extraction (TESE) allows us to extract sperm directly from the seminiferous epithelium. After several incisions are made in the outer layer of the testicle (*tunica albuginea*), the samples are collected, sent to the embryology laboratory, and cryopreserved in a tissue bank (8). This procedure is called conventional TESE (cTESE) (9). Mature spermatozoa are then used for various procedures of medically assisted reproduction (MAR), predominantly intracytoplasmic sperm injection (ICSI). MAR (ICSI and TESE-ICSI) has effectively employed freezing and thawing of mature sperm (9).

Non-obstructive azoospermia (NOA) is a more serious condition, characterized by a failure of spermatogenesis within the testis. NOA exhibits different histological pictures of spermatogenesis (and its disruption): hypospermatogenesis, spermatogenic (maturation) arrest (MA), Sertoli cell only phenotype (SCO), tubular fibrosis, and mixed atrophy of seminiferous tubules. NOA can be caused by varicocele, medications, radiation, toxins, Klinefelter syndrome, and a lack of hormones that stimulate spermatozoa production (usually after chemotherapy or due to hormonal disbalance) (10). In these cases, microsurgical testicular sperm extraction (mTESE) is employed (11). Using a specialized high-magnification microscope, an andrologist examines every inch of the exposed seminiferous tubules to check for any pockets of spermatozoa. After that, a few small tissue samples are taken out for analysis. In NOA, spermatogenesis is frequently not homogeneous throughout the testes; also, the majority of seminiferous tubules exhibit neither regular stratification of seminiferous epithelium nor a high number of spermatogenic cells but rather small, thin tubules devoid of spermatozoa or any other type of normal spermatogenesis. An accurate microdissection allows seminiferous tubules with complete spermatogenesis to be distinguished more easily. Under a microscope, these tubules appear bigger and more opaque than the surrounding tubules (12). They can be quickly examined under an inverted microscope to check for the presence of spermatozoa.

Salvage TESE (cTESE or mTESE) is a surgical method of treating male infertility performed after a failed sperm extraction procedure (13). Significant predictors of its outcome are age, testicular volume, luteinizing hormone, follicle-stimulating hormone, hypospermatogenesis, Sertoli cell-only phenotype, and spermatogenic arrest (14). These predictors may help andrologists in making clinical decisions and reducing needless reoperations. Salvage mTESE (if performed in well-established andrological centers) recovered spermatozoa in 49.2% of the patients with previously negative cTESE results (15). It is a safe procedure without any significant early post-surgical problems (15).

Male infertility is a growing issue in terms of population. For people with diagnosed azoospermia, the only chance for biological child conception is through sperm retrieval treatments. In these cases, reproductive tissue banks have an important role in preserving samples and allowing patients to prepare appropriately for the next steps of MAR.

## The foundation and activities of the Testicular Tissue Bank at UHC Zagreb

Since the establishment of the Testicular Tissue Bank at the UHC Zagreb in 2013, all patients who have undergone testicular tissue collection previously consulted an andrologist. Approximately 9% of them were oncofertility patients who stored their samples to preserve fertility. In other patients, the cause of infertility was the male factor. Most patients were diagnosed with NOA, with only a small number being diagnosed with OA. Each patient underwent a complete diagnostic work-up, including physical examination, semen analysis (at least two samples), hormone analysis (follicle stimulating hormone, luteinizing hormone, prolactin, testosterone, inhibin-B), genetic testing (karyotype, Y-chromosome microdeletions, cystic fibrosis transmembrane conductance regulator gene /CFTR/testing), ultrasound (testis consistency and volume), microbiological testing (ejaculate, urethral smear; hepatitis, HIV), and other relevant examinations. On the basis of diagnostic results, it was decided whether the tissue should be collected by cTESE or mTESE. mTESE has only been applied in our institution since 2022 in patients with the most complex diagnoses (genetic disorders or previously unsuccessful cTESE procedures). The salvage treatment (predominantly by mTESE) was performed only in those patients who had undergone unsuccessful spermatozoa retrieval attempts. Overall, 12% of patients had genetic disorders: microdeletions of the azoospermia factor (*AZF*) in the Yq region (50% of patients with genetic disorders), Klinefelter's syndrome (24%), *CFTR* gene mutations (11%), and other genetic disorders (15%).

### Collection, processing, and slow programmed freezing of testicular tissue

Testicular tissue samples were collected in the operating theater of the Department of Urology (UHC Zagreb) by using cTESE (16,17) or mTESE (11). Briefly, upon the excision, several pieces were taken from different parts of the testis and immediately immersed into a transport medium (Origio Sperm Wash, Malov, Danemark). Whenever possible, a bilateral biopsy was performed. Afterwards, each sample was divided into two parts: one was immersed in 1 mL of a freezing medium (Quinn's Advantage Sperm Freezing Medium, Sage Media, Trumbull, CT, USA) and intended for slow programmed freezing. The other was immersed in 10% formalin or Bouin's solution and intended for a pathohistological analysis. The testicular samples collected for cryopreservation were then subjected to slow programmed freezing using a Nicool LM 10 freezing device (Air Liquide, Paris, France). During the first 5 min, the samples were cooled down to -60 °C, then exponentially to -120 °C in the following 50-60 min. Finally, the frozen samples were stored in liquid nitrogen containers until the tissue was distributed to the selected IVF laboratory and microdissectioned for the ICSI procedure.

### Pathohistological analysis

**Figure 1:**
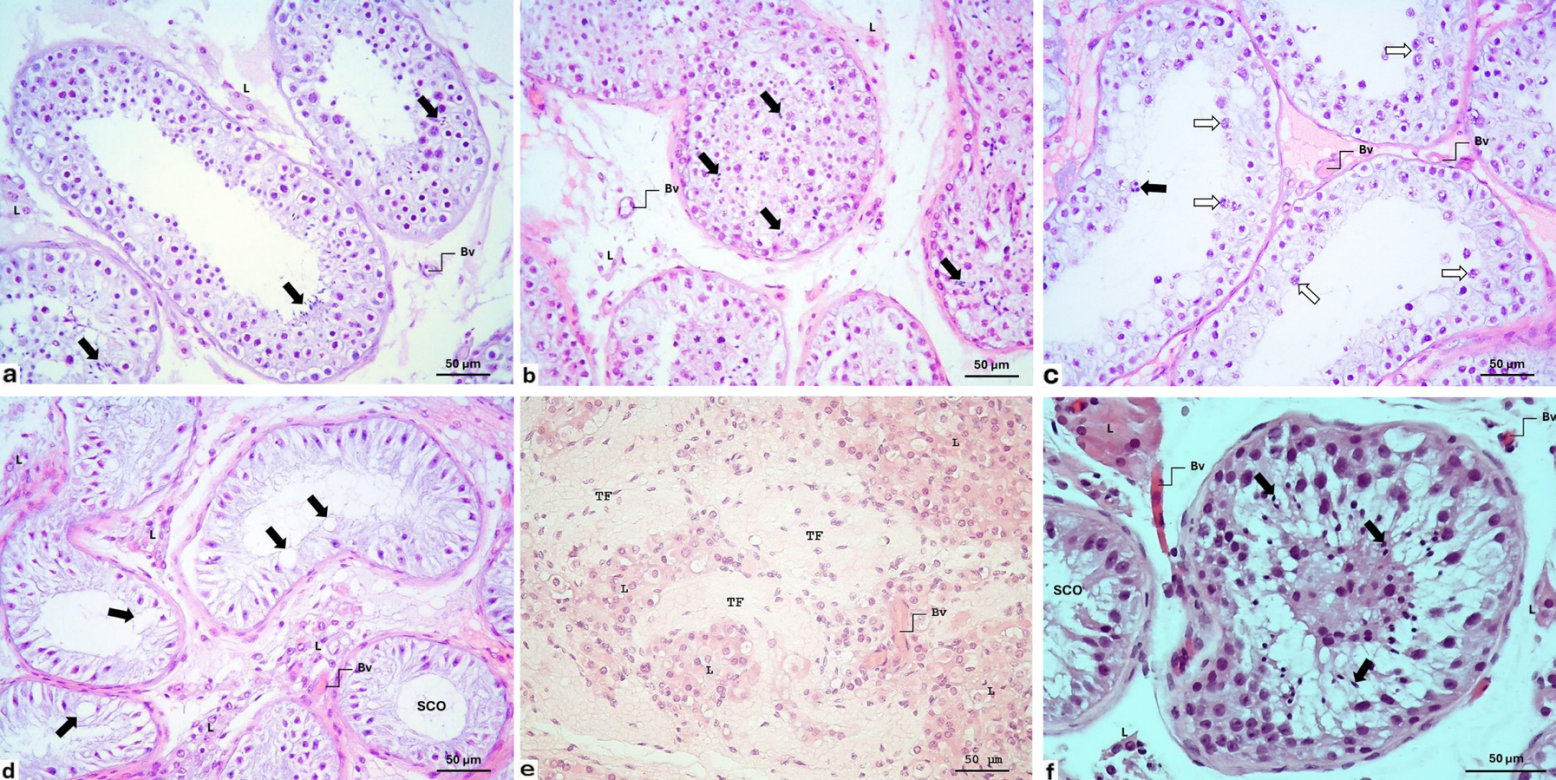
Patterns of spermatogenesis in azoospermic patients. **(A)** Full spermatogenesis. Seminiferous tubules are lined with the epithelium, showing all six spermatogenesis stages. The accumulations of late spermatids and spermatozoa are indicated by arrows. Within the loose connective tissue of the testis interstitium are clusters of Leydig cells (**L**) and some small blood vessels (Bv). Hemalaun & eosin stain (HE), ×200. **(B)** Hypospermatogenesis. All spermatogenic cells are visible within the seminiferous epithelium, including late spermatids and spermatozoa. However, these cells are not organized in typical spermatogenesis stages (see Figure 1A), and the epithelium stratification is irregular. Despite this, the spermatozoa retrieval rate in these patients, is rather successful (L – Leydig cells; Bv – small blood vessels). HE × 200. **(C)** Spermatogenic arrest (maturation “stop”). Spermatogenesis stops during the development of a certain spermatogenic cell type. In this case, the development stopped at round spermatids (black arrow). Moreover, many areas of seminiferous tubules demonstrate arrest at the level of primary spermatocytes (white arrow) (Bv – small blood vessels). HE × 200. **(D)** Sertoli cells-only phenotype (SCO), previously known as Sertoli cells-only syndrome. In this case, the seminiferous epithelium consists exclusively of Sertoli cells. Since this disruption of spermatogenesis has multiple genetic causes (which result in the same phenotype), a new nomenclature has been introduced instead of Sertoli cells-only syndrome. Due to spermatogenic cell loss, there are huge vacuoles (arrow) in Sertoli cells cytoplasm (L – Leydig cells; Bv – small blood vessels). HE × 200. **(E)** Tubular fibrosis. Seminiferous tubules are devoid of seminiferous epithelium and transformed into connective tissue strands. Between completely fibrosed tubules are abundant clusters of Leydig cells (**L**) (Bv – blood vessels). HE × 200. **(F)** Mixed atrophy of seminiferous tubules. This spermatogenesis disorder comprises a combination of seminiferous tubules that display various histological patterns of spermatogenesis (see above). In this case, a tubule with hypospermatogenesis (where all types of spermatogenic cells are present) is seen together with a tubule that is lined only by Sertoli cells [SCO]) (arrow indicates late spermatids and spermatozoa; L – Leydig cells; Bv – small blood vessels). HE × 200.

Testicular tissue samples intended for a pathohistological analysis were fixed in 10% formalin or Bouin's fluid immediately after excision. Afterwards, the usual tissue fixation was performed, including gradual tissue dehydration using ethanol, clearing with xylene, and fitting into paraffin blocks. The blocks were cut on a manual rotary microtome Leitz 1512 (Leitz, Riedau, Austria) into 4-μm thick sections stained with hematoxylin and eosin (H&E) and prepared for histological analysis. The microscopic analysis was performed with an Eclipse E600 microscope (Nikon, Tokyo, Japan). Images were made with an Axiocam digital camera (Carl Zeiss, Jena, Germany). Spermatogenesis in the prepared tissue slides was scored according to the modified Johnsen scoring system (17,18). The spermatogenesis status included the following histological pictures: complete/full spermatogenesis, hypospermatogenesis, spermatogenic arrest, Sertoli cell only phenotype (SCO), tubular fibrosis, or mixed atrophy of seminiferous tubules (a combination of the histological pictures mentioned above) (Figure 1).

### The major testicular banking outcomes at UHC Zagreb

**Figure 2:**
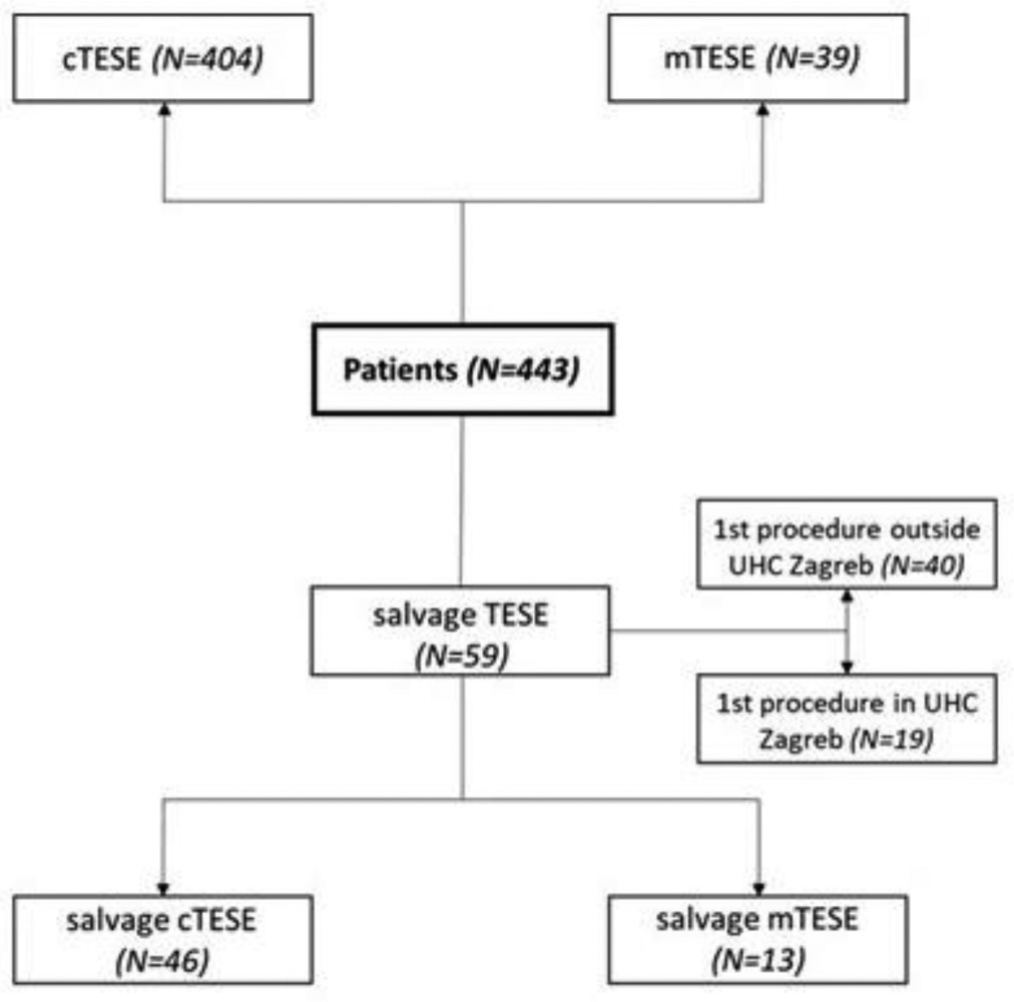
A flowchart of patients included in the Testicular Tissue Bank. Our bank stored the testicular tissue of 443 patients: 404 underwent the conventional testicular sperm extraction (cTESE), 39 underwent microsurgical TESE (mTESE), 59 underwent salvage treatment (in 40 of them, the previous sperm extraction procedure was performed in an external institution, and in 19 it was performed in the University Hospital Center (UHC) Zagreb. The salvage treatment was performed with cTESE in 46 patients and with mTESE in 13 patients.

**Figure 3:**
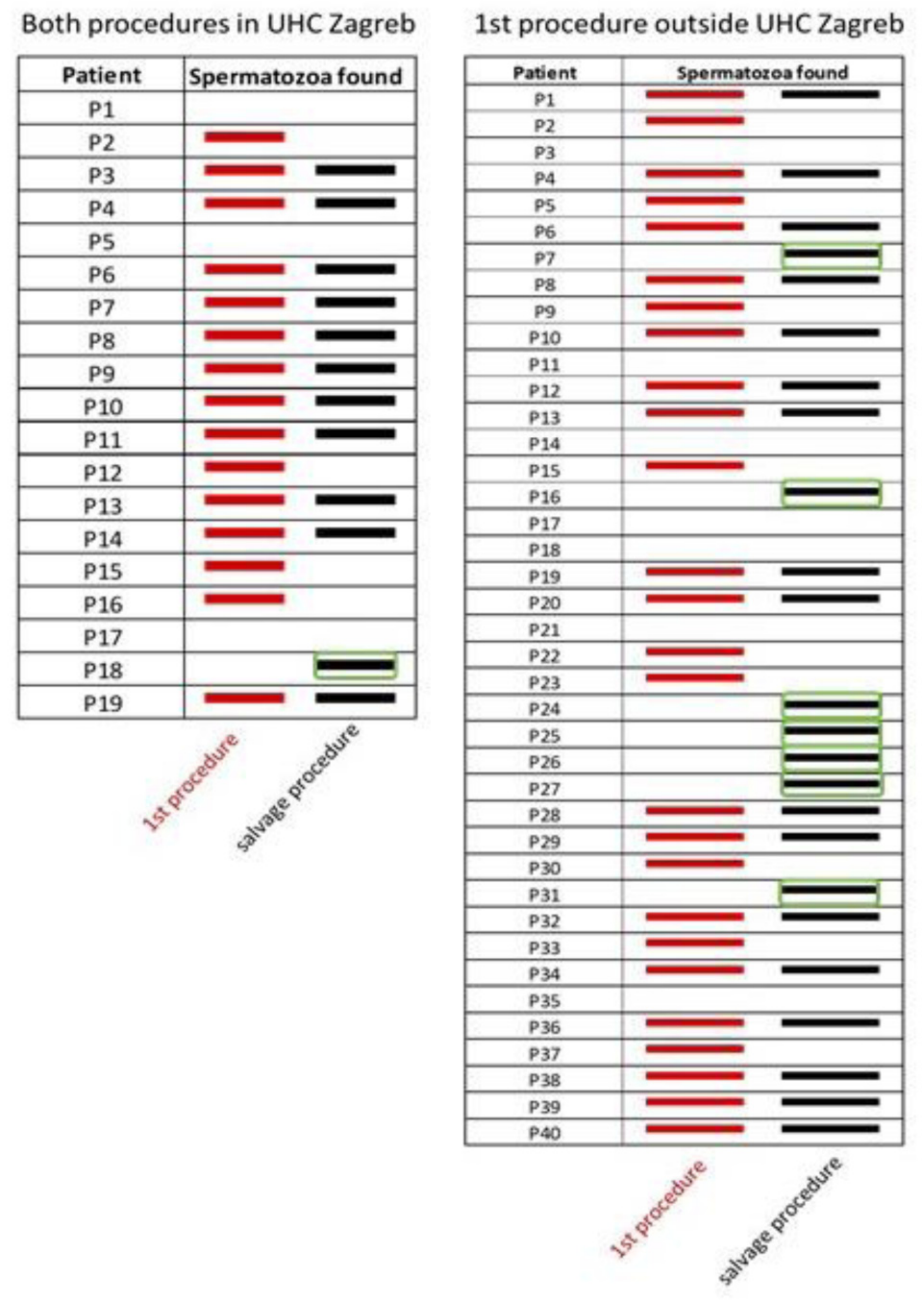
The outcome of the salvage procedure compared with the previous testicular sperm retrieval attempt. A successful sperm retrieval attempt is indicated by a red or black bar, depending on whether the spermatozoa were found in the first or salvage procedure. In 21 out of 59 patients who underwent a salvage TESE procedure, the result of the salvage procedure did not match the result of the first testicular sperm retrieval attempt. In 8 patients, spermatozoa were found only during the salvage procedure (circled).

Over the last ten years, our bank has stored the samples from 443 patients (Figure 2). Overall, 404 patients underwent cTESE, 39 patients underwent mTESE, while 59 patients underwent salvage treatment using cTESE or mTESE. In these patients, the previous sperm extraction procedure (PESA, TESA, or cTESE) was carried out either in an outside institution (40 patients) or in our institution (19 patients). In 21 out of 59 patients (36%) who underwent a salvage TESE procedure, the outcome did not match the result of the previous retrieval attempt. Thirteen (22%) patients experienced an inferior outcome (ie, sperm was not found) and 8 (14%) experienced a superior outcome. A superior outcome was more common in patients who underwent the previous sperm extraction in an outside institution and the salvage procedure in UHC Zagreb (Figure 3). According to pathohistological analysis, 17% of patients were diagnosed with complete spermatogenesis or hypospermatogenesis, 11% with spermatogenic arrest, 48% with mixed atrophy of seminiferous tubules, and 24% with Sertoli cell-only phenotype or tubular fibrosis. Overall, testicular spermatozoa were found in 58% of patients, which made them suitable candidates for a ICSI procedure.

## Demographic significance of testicular tissue banking

The results obtained at the Testicular Tissue Bank during the last ten years best indicate its significance in Croatian reproductive medicine: testicular tissue from more than 400 patients has been stored as the only option for fertility preservation and infertility treatment. Overall, the sperm retrieval success rate in our patient population is 58%, a rate comparable with well-recognized infertility world centers (11,19-21). The vast majority of our patients underwent the cTESE procedure, while less than 10% of them underwent mTESE. This is not surprising as mTESE has only been used at UHC Zagreb since the beginning of 2022. Over time, we are expected to gain more experience in selecting patients for this procedure.

It is challenging to conclude on the success of the salvage treatment because only a small number of our patients underwent the procedure. However, more than a third of these patients experienced a superior outcome compared with the first procedure. An inferior outcome may be the result of damage to the testicular tissue during the former procedure. Removing significant amounts of testicular tissue when collecting multiple testicular biopsies may interrupt blood supply underneath the tunica albuginea. This can lead to testicular devascularization and testicular atrophy (22). Furthermore, a reduced testosterone level after the first TESE procedure has also been described (23,24). Several mechanisms may be responsible for the decline in serum testosterone, including loss of testicular tissue removed during surgery, surgical trauma, or inflammation (22). Testosterone level also decreases with age (25). An improved outcome was observed in fewer patients, primarily when the salvage procedure was performed in our institution and the first procedure outside the UHC Zagreb. This may be because the Department of Urology of UHC Zagreb has many years of comprehensive experience in collecting testicular tissue (26), more than any other institution in Croatia.

In conclusion, considering that one of the leading causes of infertility is the male factor, the results obtained from the Testicular Tissue Bank activity confirm its important role in improving the demographic picture of Croatia. Since there is no standard IVF registry in Croatia, the exact number of children born using the TESE/ICSI procedure is unknown. However, according to our information, during the last ten years more than 100 children have been born after this treatment. In addition to its important role in infertility treatment, this method offers the possibility of preserving fertility in oncological patients.
